# CREB signaling activity correlates with differentiation and survival in medulloblastoma

**DOI:** 10.1038/s41598-021-95381-0

**Published:** 2021-08-09

**Authors:** Inna Armandari, Walderik W. Zomerman, Sabine L. A. Plasschaert, Marlinde J. Smit, Tosca. E. I. Martini, Eduardo S. de Camargo Magalhães, Shanna M. Hogeling, Geesina C. Rozema-Huizinga, Harm J. Lourens, Tiny G. J. Meeuwsen-de Boer, Frank J. G. Scherpen, Eveline S. J. M. de Bont, Sophia W. M. Bruggeman

**Affiliations:** 1 Department of Ageing Biology/ERIBA, University Medical Center Groningen, University of Groningen, Antonius Deusinglaan 1, 9713 AV Groningen, the Netherlands; 2 Department of Histology and Cell Biology, Faculty of Medicine, Public Health and Nursing, Universitas Gadjah Mada, Sekip Utara, 55281 Yogyakarta, Indonesia; 3 Department of Pediatric Oncology and Hematology/Pediatrics, University Medical Center Groningen, University of Groningen, Hanzeplein 1, 9700 RB Groningen, the Netherlands; 4 Princess Máxima Center for Pediatric Oncology, Lundlaan 6, 3584 EA Utrecht, The Netherlands; 5 Glial Cell Biology Laboratory, Biomedical Sciences Institute, Federal University of Rio de Janeiro, Rio de Janeiro, 21949-590 Brazil; 6 Department of Experimental Hematology, University Medical Center Groningen, University of Groningen, Hanzeplein 1, 9700 RB Groningen, the Netherlands; 7 Department of Pathology and Medical Biology, University Medical Center Groningen, University of Groningen, Hanzeplein 1, 9700 RB Groningen, the Netherlands

**Keywords:** Neurogenesis, Adult neurogenesis, Developmental neurogenesis, Cancer, CNS cancer

## Abstract

While there has been significant progress in the molecular characterization of the childhood brain cancer medulloblastoma, the tumor proteome remains less explored. However, it is important to obtain a complete understanding of medulloblastoma protein biology, since interactions between proteins represent potential new drug targets. Using previously generated phosphoprotein signaling-profiles of a large cohort of primary medulloblastoma, we discovered that phosphorylation of transcription factor CREB strongly correlates with medulloblastoma survival and associates with a differentiation phenotype. We further found that during normal cerebellar development, phosphorylated CREB was selectively expressed in differentiating cerebellar granule neuron progenitor (CGNP) cells. In line, we observed increased differentiation in CGNPs treated with Forskolin, Bmp6 and Bmp12 (Gdf7), which induce CREB phosphorylation. Lastly, we demonstrated that inducing CREB activation via PKA-mediated CREB signaling, but not Bmp/MEK/ERK mediated signalling, enhances medulloblastoma cell sensitivity to chemotherapy.

## Introduction

Medulloblastoma is a malignant tumor of the cerebellum that accounts for approximately fifteen to twenty percent of all pediatric brain tumors^1^. Based on transcriptional and (epi)genetic profiling studies, medulloblastoma can be divided into multiple different subtypes with different biological and clinical characteristics that are part of four main consensus subgroups: Wingless (WNT), Sonic hedgehog (SHH), Group 3, and Group 4^2^. A less explored aspect of medulloblastoma research is the tumor proteome. However, understanding medulloblastoma protein biology is important, since interactions between proteins are potentially targetable^3^.

Recent work from our group has shown that despite large genetic and transcriptional heterogeneity, medulloblastoma is characterised by two main phosphoprotein-signaling profiles, one of which resembling MYC-like signaling that can be targeted using protein synthesis and cell cycle inhibitors^4^. We now focused on individual phosphoproteins and report that the phosphorylation status of the CREB peptide (pCREB Ser^133^) strongly correlates with medulloblastoma survival. CREB (cAMP-responsive element (CRE) binding protein) belongs to the family of basic leucine zipper (bZIP) transcription factors^5^. It has a central position downstream various growth factor signaling pathways and orchestrates cell survival, growth, and differentiation in a number of cell types, including embryonic and adult neural progenitors^5,6^. In canonical CREB signaling, CREB becomes activated through phosphorylation of Serine 133 in response to the cAMP-dependent protein kinase A (PKA) pathway^7^. In turn, CREB Serine 133 phosphorylation (phospho-CREB) promotes its interaction with transcriptional co-activator CREB-binding protein (CREBBP/CBP) or homolog p300 (encoded by the *CREBBP* and *EP300* genes, respectively)^8,9^. Subsequently, the CREB-CREBBP/p300 transcriptional complex activates CRE-elements of target genes^10^. In addition, several other kinases can target CREB non-canonically such as calmodulin-dependent kinases II/IV, pp90^rsk^, MSK-1, MEK/ERK and AKT, underscoring the complexity of CREB-CREBBP/p300 activity^5,11,12^.

Interestingly, CREB plays a role in the differentiation of cerebellar granule neuron progenitors (CGNPs), a major cerebellar progenitor cell type that is believed to be cell-of-origin for SHH and Group 3 medulloblastoma^6,13–17^. Immature CGNPs do not express phospho-CREB. However, when they initiate differentiation into mature granule neurons, active CREB levels increase^6^. Therefore, we speculate that tumors exhibiting relatively high pCREB Ser^133^ levels have a more differentiated phenotype, which is frequently associated with increased sensitivity to therapy and thus improved outcome^18^. In line, we found enrichment for differentiation-related processes in tumors expressing high pCREB Ser^133^. This prompted us to consider CREB modulation as a therapeutic strategy and explore different approaches to induce CREB phosphorylation. We found that PKA-mediated CREB signaling, but not Bmp/MEK/ERK mediated signalling, enhances medulloblastoma cell sensitivity to Etoposide, suggesting that canonical CREB pathway activation may increase the impact of chemotherapy.

## Methods

### Cell culture and lentiviral transductions

hTERT-immortalized retinal pigmented epithelium cells (RPE-1) were purchased from the ATCC. SHH and Group 3 medulloblastoma cell lines UW426 (SHH) and MED8A (Group 3) were kindly provided by Dr. Michael S. Bobola (Seattle Children’s Hospital Research Institute, USA) and Dr. Till Milde (German Cancer Research Center (DKFZ), Heidelberg, Germany), respectively. Cell lines were cultured in DMEM (Life Technologies) supplemented with 10% fetal bovine serum (Gibco) and 100 U/ml Penicillin–Streptomycin (Life Technologies).

Murine neural progenitor cells (NPCs) were isolated from dissected neonatal C57/BL6 subventricular zone as described previously^19^. NPCs were maintained as monolayers in culture media containing DMEM/F12 supplemented with 1% N2 (Life Technologies), human EGF and basic-FGF (20 ng/mL) (PeproTech), and with addition of Laminin (1 μg/mL) (Sigma). NPCs were seeded at a density of 50.000 cells/well into 12 wells plates (Greiner).

CGNPs were harvested from postnatal day 7 (P7) cerebella of wild-type C57/BL6 mice. Dissected cerebella from one litter (males and females) were pooled and dissociated using a Papain kit following the manufacturer’s instructions (Worthington). In total, four different litters were used. Single cells were resuspended in DMEM/F12 supplemented with 1% N2, 1,5% glucose, 5 mM HEPES (Gibco) and 250 ng/mL recombinant mouse Shh (R&D systems), followed by filtration through a 40 μm cell strainer. 500.000 or 80.000 cells were seeded into poly-D-Lysine (100 μg/mL, Sigma) pre-coated 12-well plates (Greiner), or 8-well slide chambers (Ibidi), respectively.

Lentivirus was generated as described previously^4^. MED8A cells were transduced with mCherry coupled pLKO.1 Mission shRNA vectors against *CREB1* (shCREB1.1 and shCREB1.2), *CREBBP* (shCREBBP.1 and shCREBBP.2) and *EP300* (shEP300) (target sequences in Supplementary Table S2). A vector containing a scrambled hairpin (shSCR) was used as control. Sensitivity to chemotherapy was determined as area under the curve (AUC) after Etoposide treatment (0–10 μM) to get comparable evaluation across treated knockdown cells^20^.

All experiments involving animal material were approved by the Institutional Animal Care and Use Committee of the University Medical Center Groningen, the Netherlands, in adherence with the ARRIVE guidelines^21^.

### Cell culture assays

Cell viability assays were performed using WST-1 cell viability assays (Roche) as described previously^4^. Briefly, RPE-1, UW426, and MED8A cells were seeded in sextuple in a 96-well plate at a density of 10.000 cells/well and treated with chemotherapeutic agent Etoposide (0–10 μM for RNAi-treated MED8A; 3 μM for RPE-1 and UW426; and 0.5 μM for MED8A co-treatments), protein kinase A (PKA)-activator Forskolin (FSK) (3–5 μM) (Sigma), Bmp6 and Bmp12 (250 ng/mL) (PeproTech) or combination of Etoposide and CREB inducing agents 24 h post-seeding. Optical density was measured after 48 h treatment on a microplate reader at 450 nm.

For protein analysis, RPE-1, UW426, or MED8A cells were seeded into 6-well plates (Greiner) at a density of 250.000 cells/well. Cells were serum starved for 24 h prior to treatment. Cells were pre-treated using PKA inhibitor, H89 hydrochloride (20 μM) (Cell Signaling) or MEK inhibitor, PD98059 (25 μM) (Sigma) for 30 min or 1 h, respectively. Subsequently, cells were treated with FSK (5 μM), Bmp6 or Bmp12 (250 ng/mL) for 45 min, lysed in RIPA buffer, and analyzed by Western blotting.

NPC differentiation was induced using DMEM/F12 supplemented with 2% serum in the presence or absence of CREB blocker Naphtol AS-E phosphate/KG-501 (3 μM) (Sigma) or DMSO as control for 48 h, followed by immunofluorescence staining for differentiation markers.

CGNP differentiation was induced by treatment with FSK (5 μM) (Sigma), Bmp6 or Bmp12 (PeproTech) in CGNP culture media. CGNPs were fixed at 1 day in vitro (DIV1) for mRNA isolation and subsequent quantitative RT-PCR, or at DIV4 for immunofluorescence staining (see Supplementary file).

### Western blotting

Cells were lysed in RIPA buffer supplemented with cOmplete and PhosSTOP (Roche). Protein concentrations were determined using the BCA protein assay kit (Thermo Fisher). Proteins were separated by SDS-PAGE (BioRad) and blotted onto PVDF membrane using Trans-Blot Turbo method (BioRad). Membranes were incubated with monoclonal antibodies for phospho-CREB (1:2000), CREBBP (1:1000), phospho-ERK (1:1000) (Cell Signaling), and p300 (1:1000) (Santa Cruz). Histone H3 (1:1000), total CREB (1:2000), total ERK (1:1000) (Cell Signaling), and GAPDH (1:20,000) (Fitzgerald) were used as loading control. Secondary antibodies were goat anti-rabbit and goat anti-mouse HRP-conjugated (1:2000) (Dako). In some cases, membranes were stripped and re-probed (Thermo Fisher). Protein expression levels were measured using the ChemiDoc MP system (BioRad).

### Immunofluorescence staining

CGNPs and NPCs were fixed with 4% formaldehyde (Sigma), permeabilized with 0.1% Triton X-PBS, and blocked with 5% normal goat serum (Thermo Fisher). Primary antibodies were GFAP (1:400) (Dako) and Nestin (1:1000) (BD Biosciences) for NPCs, and phospho-CREB (1:1000) (Cell Signaling), NeuN (1:1000), Doublecortin (1:500) (Abcam) for CGNPs. Secondary antibodies were Alexa Fluor 488 or Alexa Fluor 568 (1:500) (Invitrogen). Cells were counterstained with DAPI and imaged on a Leica inverted fluorescence microscope.

Whole brains from P7 and P30 wild-type C57/BL6 mice were fixed with 4% formaldehyde. Brains were cryoprotected with a sucrose gradient (10%, 20% and 30% sucrose in PBS) and sagitally embedded in Tissue-Tek OCT (Sakura). 6–10 μm cryosections were generated on a Leica cryostat. Antigen retrieval was performed using Citrate buffer (100 mM, pH 6.0). Immunolabelings were performed as for NPCs and CGNPs. Primary antibodies were phospho-CREB (1:1000), total CREB (1:1000) (Cell Signaling), BMP6 (1:1000) and BMP12 (1:1000) (Abcam). Slides were mounted using Vectashield (Vector Laboratories), imaged on a Leica TCS SP8 confocal microscope and analysed using Fiji^22^.

### Survival curves

Average levels of pCREB Ser^133^ peptide phosphorylation, and average levels of *CREBBP* and *EP300* mRNA expression, were calculated for the complete cohort to determine a threshold: all samples with phosphorylation or mRNA expression levels lower or higher than the threshold were assigned as Below average (dashed blue lines) or Above average (solid red lines), respectively. For molecular subgroup analysis, the average levels of pCREB Ser^133^ peptide phosphorylation were calculated for each subgroup. Kaplan–Meier curves were generated based on below (or above) pCREB Ser^133^ peptide phosphorylation level and mRNA expression level for overall survival, and p-values were calculated using the log-rank (Mantel-Cox) test. All Kaplan–Meier curves and log-rank tests were generated using Prism 8 (GraphPad). For medulloblastoma primary samples, peptide phosphorylation arrays and gene expression profiling, see Supplementary info.

### Statistical analysis

Prism 8 (GraphPad) was used to prepare the charts and perform statistical analyses. Data were analyzed by unpaired *t*-test or ordinary one-way ANOVA, as indicated in the figure legends. *P*-values < 0.05 were considered significant (*p < 0.05, **p < 0.01, ***p < 0.001, ****p < 0.0001).

### Ethics approval

All applicable national and institutional guidelines for the care and use of animals were followed. All animal experiments were approved by the Institutional Animal Care and Use Committee of the University of Groningen (IACUC-RUG) (IvD 17813–01-001).

## Results

### CREB activity correlates with overall survival in pediatric medulloblastoma patients

**Figure 1:**
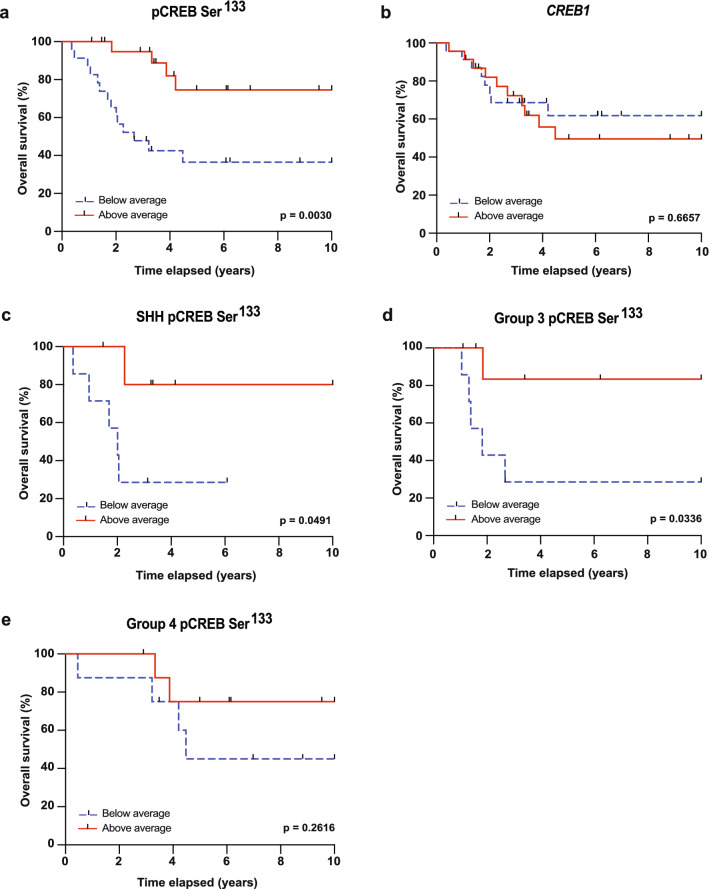
CREB phosphorylation status correlates with medulloblastoma survival. **(a,b)** Kaplan–Meier overall survival curves of medulloblastoma patients grouped by below average (dashed blue line) and above average (solid red line) based on (**a**) pCREB Ser^133^ peptide phosphorylation level; and (**b**) *CREB1* mRNA expression level. (**c–e)** Kaplan–Meier overall survival curves of (**c**) SHH (n = 13), (**d**) Group 3 (n = 15), or (**e**) Group 4 (n = 17) medulloblastoma patients grouped by below average (dash blue line) and above average (solid red line) pCREB Ser^133^ peptide phosphorylation level. P-values were determined using a log-rank (Mantel-Cox) test, and p < 0.05 was considered significant. For patient and clustering data, see Supplementary Table S1.

To identify phosphoproteins correlating with medulloblastoma outcome, we analyzed the relationship between individual phosphorylated peptides from our previously generated medulloblastoma phosphoprotein-signaling profiles, with overall survival^4^. We found that patients with relatively low levels of the peptide representing the phosphorylated (active) form of the transcription factor CREB (pCREB Ser^133^) had an extremely poor prognosis, indicating that high CREB activity correlates with overall survival (Fig. 1a). Interestingly, such relationship was not found when comparing *CREB1* mRNA levels with survival (Fig. 1b), suggesting that the role of CREB in medulloblastoma is confined to the protein compartment.

We next questioned if the correlation between pCREB Ser^133^ levels and survival was subgroup specific. Hereto, we split the cohort by molecular subgroup, which notably leads to relatively small samples sizes (SHH, n = 13; Group 3, n = 15; Group 4, n = 17)^23^. We found that levels of pCREB Ser^133^ were particularly predictive for outcome in SHH and Group 3 medulloblastoma, whereas in Group 4 medulloblastoma only a trend was observed (Fig. 1c–e). Strikingly, relative pCREB Ser^133^ peptide phosphorylation and *CREB1* mRNA levels were highest in Group 4 patients (Supplementary Fig. S1), suggesting that pCREB Ser^133^ levels are predictors of outcome in specific contexts. Together, these results suggest an important role for CREB activity in the prognosis of medulloblastoma.

### The CREB transcriptional complex is linked to medulloblastoma survival

**Figure 2:**
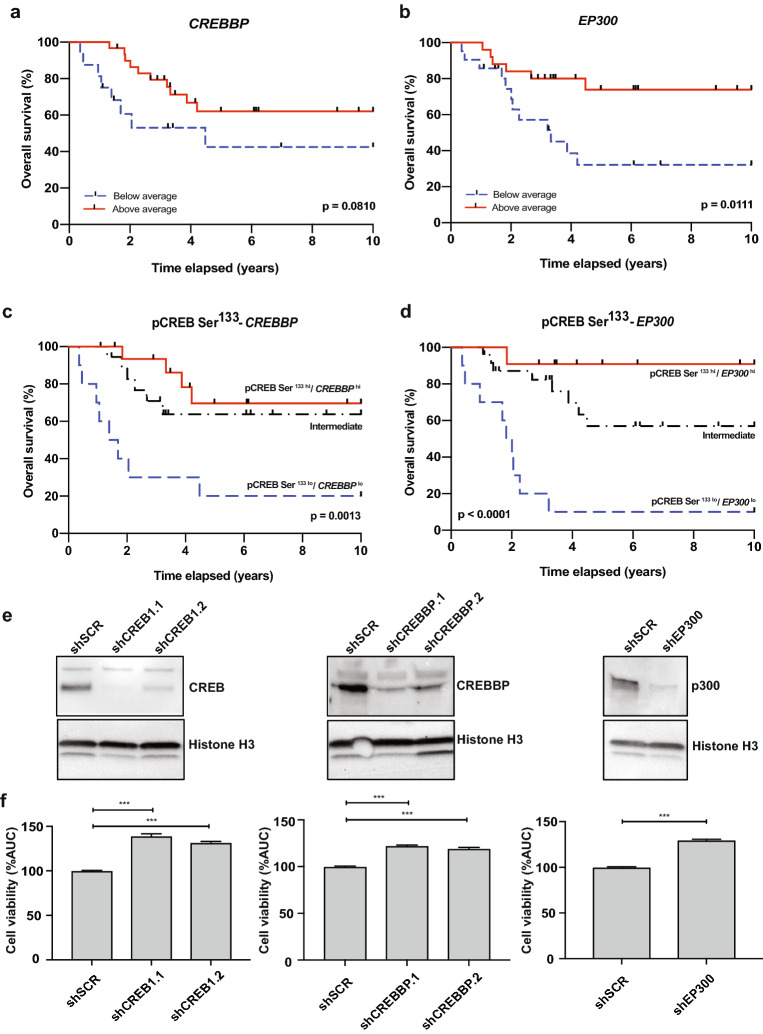
The CREB transcriptional complex is associated with medulloblastoma survival. **(a,b)** Kaplan–Meier overall survival curves of medulloblastoma patients (n = 49) grouped by below average (dashed blue line) and above average (solid red line) based on (**a**) *CREBBP* mRNA expression level, and (**b**) *EP300* mRNA expression level. **(c**,**d)** Kaplan–Meier overall survival curves of medulloblastoma patients (n = 49) grouped by average **(c)** pCREB Ser^133^ peptide phosphorylation level and *CREBBP* mRNA expression level, or **(d)** pCREB Ser^133^ peptide phosphorylation level and *EP300* mRNA expression level. Groups consisted of samples exhibiting above average pCREB Ser^133^ (pCREB Ser^133 hi^) and either above average *CREBBP,* or *EP300* mRNA expression (*CREBBP*^hi^ or *EP300*^hi^) (solid red lines, n = 16 and n = 11, respectively); samples exhibiting below average pCREB Ser^133^ (pCREB Ser^133 lo^) and either below average *CREBBP,* or *EP300* mRNA expression (*CREBBP*^lo^ or *EP300*^lo^) (dashed blue lines, n = 10 and n = 10, respectively); and samples with other combinations (Intermediate) (dashed black lines, n = 19 and n = 24). P-values were determined using a log-rank (Mantel-Cox) test, and p < 0.05 was considered significant. For patient and clustering data, see Supplementary Table S1. **(e)** Western blots showing protein expression of CREB, CREBBP, or p300 in MED8A cells with *CREB1* knockdown (shCREB1.1 and shCREB1.2), *CREBBP* knockdown (shCREBBP.1 and shCREBBP.2), or *EP300* knockdown (shEP300) compared to control (shSCR). Histone H3 was used as loading control. All images are cropped and derived from the same blot (cut for different probes). For full images, see Supplementary file. (**f**) WST-1 cell viability assays showing the cell viability of MED8A cells after *CREB1* knockdown, *CREBBP* knockdown, or *EP300* knockdown compared to control (shSCR) upon treatment with Etoposide (concentration range, 0–10 μM) (n = 3, unpaired t-test). *AUC* area under the curve. ***p < 0.001.

Phosphorylation of CREB Serine 133 induces binding to the histone acetyltransferase CREBBP or its homolog p300, to form CREB/CREBBP or CREB/p300 transcriptional complexes^8,9^. To explore a role for CREB-complexes in medulloblastoma, we first investigated the relationship between *CREBBP* and *EP300* mRNA expression and overall survival. We found that patients expressing higher *CREBBP* and *EP300* mRNA expression had improved outcome, and this was significant for *EP300* (p = 0.0111) (Fig. 2a,b; Supplementary Fig. S2). We then grouped the medulloblastoma samples into below (or above) average pCREB Ser^133^ (pCREB Ser^133 hi/lo^), in combination with below (or above) average *CREBBP* (*CREBBP*^hi/lo^) or *EP300* (*EP300*^hi/lo^) mRNA levels, and assessed their relationship with overall survival. Samples that did not meet these criteria were arbitrarily assigned to the Intermediate group. We found no difference in overall survival between patients from the Intermediate group and the pCREB Ser^133/hi^ *CREBBP/EP300*^*hi*^ groups (Fig. 2c,d). Remarkably, we did observe highly significant correlations between poor outcome and low pCREB Ser^133^ levels in combination with either low *CREBBP* (p = 0.0013), or *EP300* (p < 0.0001) mRNA expression (Fig. 2c,d).

We then functionally confirmed that CREB complex members impact on medulloblastoma cell survival. Hereto, we performed knockdown of either *CREB1*, *CREBBP*, or *EP300* in MED8A cells, an in vitro model for Group 3 medulloblastoma (Fig. 2e) and subsequently determined their sensitivity to the chemotherapeutic agent Etoposide. In agreement with our findings in patients, we observed that loss of CREB-complex expression increased medulloblastoma cell viability (Fig. 2f). These data suggest that CREB/p300, and to a lesser extent CREB/CREBBP, are beneficial for medulloblastoma outcome.

### CREB-complex activity is associated with neural differentiation in tumors and developing CGNPs

**Figure 3:**
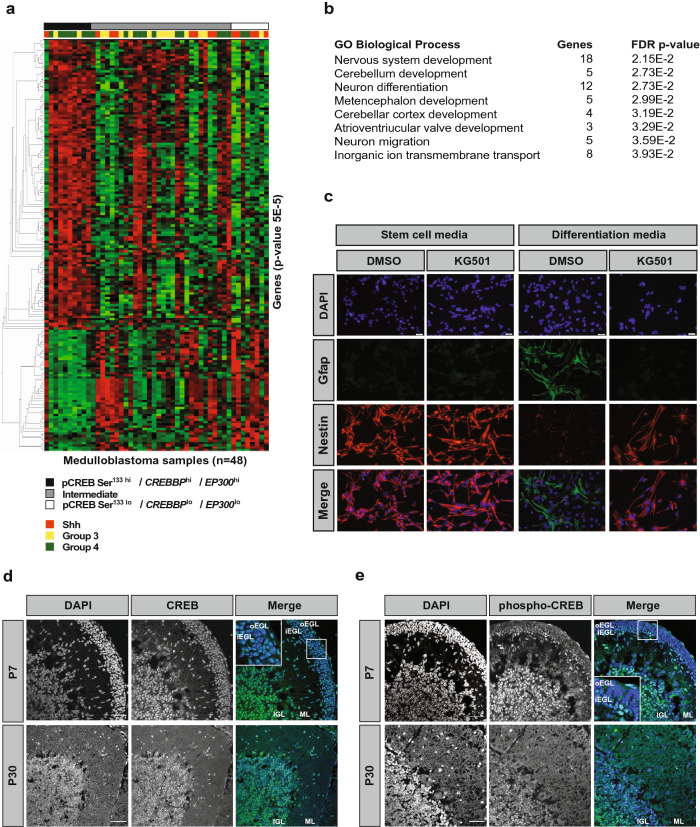
CREB-complex activity is associated with neural differentiation in tumors and developing cerebellum. **(a)** Heatmap showing the supervised hierarchical clustering of quantile normalized gene expression levels of primary medulloblastoma samples (n = 48) grouped by peptide phosphorylation and mRNA levels: pCREB Ser^133 lo^, *EP300*^lo^ and *CREBBP*^lo^ (white); pCREB Ser^133 hi^, *EP300*^hi^ and *CREBBP*^hi^ (black); or other combinations (Intermediate) (grey). Red indicates relatively high gene expression and green relatively low gene expression. Colored squares indicate molecular subgroup (Shh, red; Group 3, yellow; Group 4, green). For patient and clustering data, see Supplementary Table S1. **(b)** List of biological processes related to genes upregulated in pCREB Ser^133 hi^, *EP300*^hi^ and *CREBBP*^hi^ medulloblastoma tissue samples. **(c)** Representative fluorescent microphotographs showing DAPI (blue), Gfap (green), and Nestin (red) expression in neural stem cells treated with low dose KG-501 (3 μM) or mock (left panels), or differentiated cells with or without KG-501 (3 μM) (right panels). Scale bars indicate 500 μm. **(d,e)** Confocal images showing (**d**) CREB or (**e**) phospho-CREB expression in postnatal day 7 (P7) mouse cerebellum and P30 cerebellum. Scale bars indicate 45 μm. Insets show magnified views of CREB and phospho-CREB expression in the EGL (external granular layer). *oEGL* outer external granular layer; *iEGL* inner external granular layer; *ML* molecular layer; *IGL* internal granular layer.

It is known that CREB-complex activity is associated with neural differentiation, including in the cerebellum^6,13^. We therefore questioned if the medulloblastoma samples in our cohort with high CREB-complex activity also exhibited a differentiated phenotype, which could explain the improved outcome^18^. We again categorized the medulloblastoma samples into three groups based upon pCREB Ser^133 hi/lo^ levels, in combination with *CREBBP*^hi/lo^ and *EP300*^hi/lo^. We then took advantage of our data set of medulloblastoma transcriptomes that we had generated previously^4^, and performed supervised hierarchical clustering according to the three groups. This revealed two robust gene expression clusters that were reciprocally enriched in the pCREB Ser^133 hi^/*CREBBP*^hi^/ *EP300*^hi^ (black) or pCREB Ser^133 lo^/*CREBBP*^lo^/*EP300*^lo^ (white) groups (Fig. 3a). Samples from the Intermediate group (grey) exhibited either gene expression profile. Interestingly, subsequent functional annotation of these two gene clusters revealed that biological processes related to neuronal differentiation and development were highly enriched in the pCREB Ser^133 hi^/*CREBBP*^hi^/*EP300*^hi^ group (Fig. 3b). No enriched biological processes were found in genes enriched in the pCREB Ser^133lo^/*CREBBP*^lo^/*EP300*^lo^ group. Of note, when we specifically analyzed the expression levels of genes involved in CGNP differentiation (*i.e., ZIC* and *GRIN* genes) across the medulloblastoma cohort, we found that *ZIC2*, *GRIN2A* and *GRIN2C* were relatively high in SHH tumors, even though these tumors clustered mostly to the Intermediate and pCREB Ser^133lo^/*CREBBP*^lo^/*EP300*^lo^ group (Supplementary Fig. S3). This suggests that not all differentiation is blocked in pCREB Ser^133lo^/*CREBBP*^lo^/*EP300*^lo^ tumors.

To further explore the link between the CREB complex and differentiation, we next functionally tested if the interaction between CREB and CREBBP/p300 is required for neural differentiation using neural progenitor cells (NPCs) as an in vitro model. We induced NPC differentiation in the presence or absence of a low dose of KG501, a blocker of the interaction between CREB and CREBBP/p300^24^, and found that while NPCs rapidly induce expression of differentiation marker Gfap and lose expression of stem cell marker Nestin in the presence of serum, KG-501 treatment completely inhibits the appearance of differentiated cells (Fig. 3c). This indicates that the CREB-CREBBP/p300 interaction is required for normal NPC differentiation.

We subsequently investigated phospho-CREB expression in the putative cell-of-origin for SHH and Group 3 medulloblastoma, which is the CGNP^14,16,17^. CGNPs reside in the external granular layer (EGL) of the developing cerebellum before migrating to the internal granular layer (IGL), where they mature into granule neurons^25,26^. We examined the expression pattern of CREB and phospho-CREB in these cells in postnatal day 7 (P7) and day 30 (P30) cerebellum (Fig. 3d,e). In line with earlier findings^27^, we find that total CREB is expressed in the majority of CGNPs in all cellular layers (Fig. 3d). In contrast, phospho-CREB is selectively expressed in areas enriched for differentiating CGNPs, including the P7 inner EGL (iEGL) and IGL (Fig. 3e). The outer EGL (oEGL), where immature proliferating cells reside, is negative for phospho-CREB. These data suggest a role for active CREB in CGNP differentiation.

### CREB pathway activation induces CGNP differentiation

**Figure 4:**
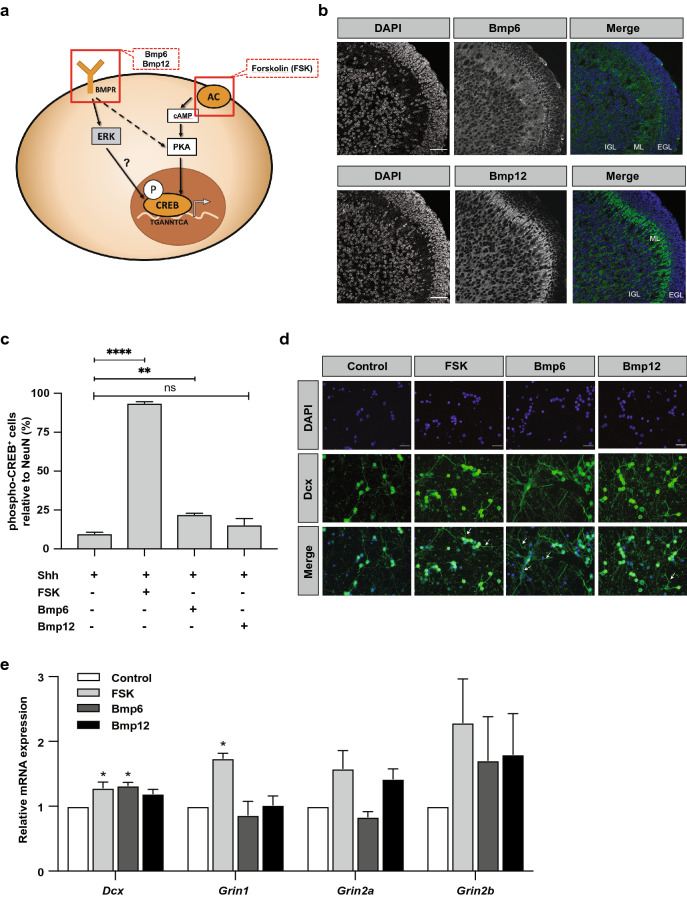
CREB phosphorylation is associated with cerebellar differentiation. **(a)** Schematic overview of selected CREB-activating pathways. FSK activates the cAMP/PKA pathway by binding to AC. Bmp6/12 likely activate the MEK/ERK pathway and possibly also PKA. *BMPR* bone morphogenetic protein receptor; *FSK* forskolin; *AC* adenylate cyclase; *cAMP* cyclic adenosine monophosphate; *PKA* protein kinase A; *ERK* extracellular signal-regulated kinase; *P* phosphorylated. **(b)** Confocal images showing Bmp6 and Bmp12 protein localization in postnatal day 7 (P7) mouse cerebellum. Scale bars indicate 45 μm. *EGL* external granular layer; *ML* molecular layer; *IGL* internal granular layer. **(c)** Quantification of phospho-CREB expression in CGNPs treated with FSK (5 μM), Bmp6 (250 ng/mL), Bmp12 (250 ng/mL), or control (n = 3, Dunnett *post-hoc* test of one-way ANOVA). **(d)** Representative fluorescent microphotographs showing DAPI (blue) and Doublecortin (Dcx) (green) expression in CGNPs treated with FSK, Bmp6/Bmp12, or control. Arrows indicating longer neurites. Scale bars indicate 500 μm. **(e)** Quantitative RT-PCR for neuronal genes *Doublecortin* (*Dcx*), and glutamate receptor subunits *Grin1, Grin2a*, and *Grin2b* in P7 CGNPs treated with FSK, Bmp6/Bmp12, or control (n = 3, Dunnett *post-hoc* test of one-way ANOVA). All charts represent mean ± SEM. *p < 0.05, **p < 0.01, ****p < 0.0001.

We then wondered if inducing CREB pathway activation would result in CREB phosphorylation and subsequent differentiation. We focused on the PKA pathway that can be stimulated by the compound Forskolin (FSK), and the MEK/ERK pathway that is responsive to Bmp (Bone Morphogenetic Protein) factors (Fig. 4a). We selected Bmp6 and Bmp12 (Gdf7) as ligands for Bmp pathway stimulation, since we found that they are overlappingly expressed with phospho-CREB at sites of CGNP differentiation (Fig. 4b)^28^.

We first tested if FSK/Bmp6/Bmp12 were able to induce phospho-CREB expression in primary CGNP cultures. We found that all three compounds could induce phospho-CREB, with FSK being the most potent (Fig. 4c). We further found that FSK/Bmp6/Bmp12 treatment induced CGNP differentiation, as shown by an increase in Doublecortin (Dcx) positive neurites and connections between cells (Fig. 4d). Moreover, we observed a significant increase in mRNA expression of differentiation markers *Dcx* and glutamate receptor subunit *Grin1* (Fig. 4e). Resuming, all three compounds induce phospho-CREB and promote CGNP differentiation, albeit to different extents, with Forskolin being the most potent except for the formation of Dcx neurites.

### Exploiting CREB pathway activation to target medulloblastoma cells

**Figure 5:**
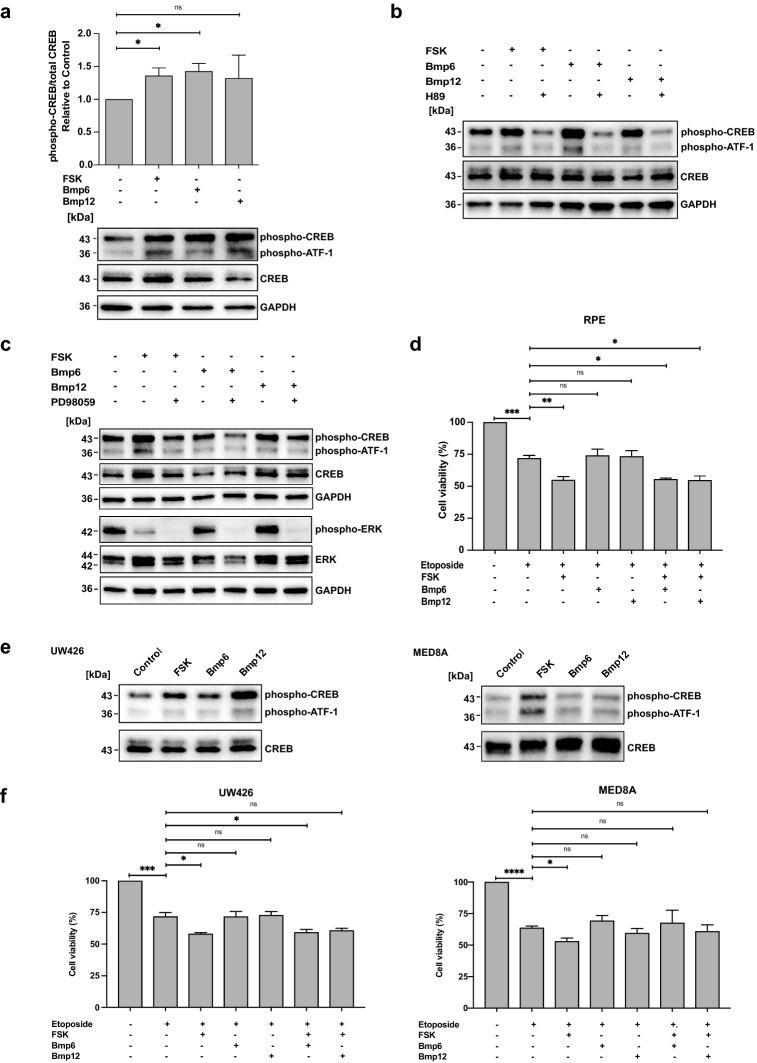
CREB pathway activation enhances chemosensitivity. **(a)** Western blot and quantification by densitometry showing phospho-CREB and total CREB expression in serum starved RPE-1 cells following treatment with FSK (5 μM), Bmp6 (250 ng/mL), Bmp12 (250 ng/mL), or control (n = 4, unpaired t-test). Images are cropped and derived from the same blot (re-probed after stripping). GAPDH is used as a loading control. **(b)** Western blot showing phospho-CREB and total CREB expression in serum starved RPE-1 cells following treatment with FSK (5 μM), Bmp6 (250 ng/mL), Bmp12 (250 ng/mL), or control in the presence or absence of PKA pathway inhibitor H89 (20 μM). Images are cropped and derived from the same blot (re-probed after stripping). GAPDH is used as a loading control. **(c)** Western blot showing phospho-CREB, total CREB, phospho-ERK and total ERK expression in serum starved RPE-1 cells following treatment with FSK (5 μM), Bmp6 (250 ng/mL), Bmp12 (250 ng/mL), or control in the presence or absence of MEK/ERK pathway inhibitor PD98059 (25 μM). Images are cropped. CREB and phospho-CREB images derived from the same blot (re-probed after stripping). For phospho- and total ERK, samples were re-loaded onto another gel. Images are derived from the same blot. GAPDH is used as a loading control. **(d)** Chart showing RPE-1 cell viability upon treatment with Etoposide (3 μM), FSK (2 μM), Bmp6/12 (250 ng/mL) for 48 h (n = 3 experiments, unpaired t-test, Dunnett *post-hoc* test of one-way ANOVA). **(e)** Western blot showing phospho-CREB and total CREB expression in serum starved UW426 (left) and MED8A (right) cells following treatment with FSK (5 μM), Bmp6 (250 ng/mL), Bmp12 (250 ng/mL), or control. Images are cropped and derived from the same blot (re-probed after stripping). **(f)** Chart showing UW426 (left) and MED8A (right) cell viability upon treatment with Etoposide (3 and 0.5 μM, respectively), FSK (2 μM), Bmp6/12 (250 ng/mL) for 48 h (n = 3, unpaired t-test, Dunnett *post-hoc* test of one-way ANOVA). All charts represent mean ± SEM. *p < 0.05, **p < 0.01. For full images of the Western blots, see Supplementary file.

We then wanted to explore if FSK/Bmp treatment can enhance medulloblastoma chemosensitivity. Since it has been suggested that FSK and Bmp6/12 pathways are (partially) non-redundant, which could translate into synergistic effects upon co-treatment, we first studied pathway usage by FSK and Bmp6/12. Hereto, we employed a cell culture model (*i.e*., RPE-1 cells) resembling normal, non-transformed human cells, to study the pathways under native conditions^29–31^. This allowed us to study CREB activity in normal, unaltered signaling pathways. We first confirmed that treatment of serum starved RPE-1 cells with FSK, Bmp6, and Bmp12 was able to induce phospho-CREB expression (Fig. 5a). Simultaneous inhibition of the PKA pathway using PKA inhibitor H89 resulted in a reduction in phospho-CREB expression in all cases, suggesting that PKA is involved in relaying both FSK and Bmp signals towards CREB (Fig. 5b, Supplementary Fig. S4a). However, while inhibition of the MEK/ERK pathway using PD98059 resulted in a significant phospho-CREB reduction after Bmp6 treatment, we only observed a trend towards inhibition for Bmp12 and FSK (Fig. 5c, Supplementary Fig. S4b,c). Unexpectedly, we found that FSK treatment also led to a reduction in phospho-ERK signal regardless of PD98059 treatment, suggesting further crosstalk and partial redundancy between the two pathways.

Finally, to test if FSK/Bmp6/Bmp12 can enhance the response to chemotherapy, we co-treated RPE-1 cells with Etoposide and FSK, Bmp6, or Bmp12. We found that cell viability after treatment with Etoposide and FSK is lower than after Etoposide treatment alone, whereas Bmp6/Bmp12 treatment had no effect (Fig. 5d). Further, we observed no synergistic effect from co-treatment with FSK and Bmps. We then repeated these experiments in SHH (UW426) and Group 3 (MED8A) medulloblastoma cells and observed that both cell lines responded to FSK/Bmp6/Bmp12 treatment with CREB phosphorylation following serum starvation, albeit at varying rates (Fig. 5e). Similar to our observations in RPE1 cells, we also found that FSK treatment increased chemosensitivity (Fig. 5f). Altogether, our results show that while FSK/Bmp6/Bmp12 are all able to induce CREB phosphorylation, only FSK-mediated CREB activation might be useful as chemotherapy adjuvant.

## Discussion

By investigating the medulloblastoma phosphoproteome, we identified relatively high levels of active (phosphorylated) CREB as a strong predictor for improved overall survival. We further found that processes related to neuronal differentiation are enriched in tumors with high CREB complex activity. This prompted us to explore the link between CREB phosphorylation and differentiation, which we hypothesized could be exploited for medulloblastoma treatment. Intriguingly, while we found that pCREB Ser^133^ levels were highest across Group 4 patients, they were most significantly associated with outcome in SHH and Group 3 patients. This suggests that CREB phosphorylation per se is not sufficient to determine outcome, but that context is important. Of note, in a study investigating alternative splicing in medulloblastoma, it was found that CREB signaling-related processes were enriched in SHH and Group 3, but not Group 4 medulloblastoma^32^. Thus, it is plausible that SHH and Group 3 medulloblastoma are intrinsically more dependent on CREB signaling and as a result have increased sensitivity to alterations in CREB activity. This may be related to the fact that SHH and Group 3 medulloblastoma originate from the same cellular compartment, as further discussed below.

### CREB-complex activity is associated with cerebellar development

The CREB-complex can induce transcription of a large number of CRE-containing genes^5^. The precise set of target genes is context dependent, implying that the overall outcome of CREB activation varies between cell types. This is exemplified by the fact that whereas several previous studies, including our own, have associated active CREB with pro-oncogenic functions, for medulloblastoma we now report the opposite^33–35^. This could originate from the neuronal context of medulloblastoma. It is known that in neural differentiation, CREB is a key driving factor and in cerebellum, CREB is associated with CGNP differentiation^6,13^. Intriguingly, these CGNPs are putative cells-of-origin for SHH and Group 3 medulloblastoma, which aligns with our finding that SHH and Group 3 survival most prominently correlates with CREB activity^14–17^. Early CGNPs undergo intense proliferation in the EGL, a secondary germinal zone^25,26^. Upon reaching maturity, they migrate inwards to form the definitive IGL of granule neurons^25^. We speculate that the CREB-complex controls this process and that its activity prevents the formation of (pre)cancerous lesions^36^. This is in agreement with observations in a *Crebbp* null mouse model, in which early postnatal *Crebbp* loss enhances medulloblastoma formation^37^. Further, patients with Rubinstein-Taybi syndrome have an increased risk of developing medulloblastoma, and *CREBBP* mutations have been found in sporadic medulloblastoma^38–40^.

Following from this, we reasoned that inducing CREB phosphorylation may be beneficial in medulloblastoma therapy, and set out to identify compounds that can activate CREB in cerebellar cells. It had been reported that CREB activation is linked to extracellular factors, including Bmp family members, in areas of CGNP differentiation^25,28,41^. In addition to Bmp6, we focused on Bmp12 (Gdf7), which is a powerful player in early CGNP specification that has not yet been studied in the context of CGNP differentiation^42^. Although we discovered that CGNPs do not express Bmp6/12 themselves, we could detect Bmp6 and Bmp12 in the direct vicinity of differentiating CGNPs expressing high phospho-CREB. This suggests that other cerebellar cells supply these factors to neighboring CGNPs. Indeed, using primary cerebellar cell cultures, we could demonstrate that ectopic Bmps induce CREB phosphorylation and CGNP differentiation, and therefore are potential therapy candidates.

### Modulating CREB-complex activity

When we started to explore CREB activation as potential medulloblastoma treatment, alongside Bmps that are believed to activate CREB via MEK/ERK signaling, we included Forskolin (FSK), a potent activator of the cAMP/PKA pathway also targeting CREB^43,44^. We reasoned that targeting CREB via alternative pathways might have a synergistic effect on tumor cell chemosensitivity. However, we found that PKA inhibition also had a negative effect on Bmp-induced CREB activation, highlighting a role for PKA in the Bmp-CREB axis^45,46^. Further, we observed a decrease in MEK/ERK phosphorylation after FSK treatment^47,48^. Thus, the Bmp and FSK pathways are partially redundant and in line with this, we found no synergistic effect of Bmp and FSK co-treatment on medulloblastoma cell chemosensitivity. In fact, while FSK treatment increased chemosensitivity in all cell lines tested, for Bmp we only found such effect in UW426 cells, possibly because Bmps induce less efficient CREB phosphorylation.

Altogether, we conclude that inducing CREB activation through the PKA but not Bmp signaling cascade might be beneficial as chemotherapy adjuvant in medulloblastoma. Forskolin seems to be a promising compound for this, especially since this drug has already been clinically tested as treatment for several human diseases including diabetes and liver fibrosis; and there is increasing interest in Forskolin as anti-cancer treatment^44,49^.

## Supplementary Information

Supplementary Information 1.

Supplementary Information 2.

## Data Availability

All data generated for this study are included in this manuscript or can be found at Mendeley Data, http://dx.doi.org/10.17632/s7j3gkd58p.1.
