# Supplementary material for: CREB signaling activity correlates with differentiation and survival in medulloblastoma
Source: Sci Rep. 2021 Aug 9;11:16077. doi: 10.1038/s41598-021-95381-0 (PMC8352923; doi:10.1038/s41598-021-95381-0)
Supplement: Supplementary file 1 — Supplementary Information 1. [file 41598_2021_95381_MOESM1_ESM.pdf]

Supplementary file

CREB signaling activity correlates with differentiation and survival in medulloblastoma

Inna Armandari, Walderik W. Zomerman, Sabine L. A. Plasschaert, Marlinde J. Smit, T.E.I. Martini, Eduardo S.C. Magalhães, Shanna M. Hogeling, Geesina C. Rozema-Huizinga, Harm J. Lourens, Tiny G.J. Meeuwsen-de Boer, Frank J.G. Scherpen, Eveline S. J. M. de Bont, Sophia W.M. Bruggeman.

<u>**Content**</u>

Supplementary methods

Supplementary Table S1: See Excel file

Supplementary Table S2: Short hairpin RNA sequences

Supplementary Table S3: Quantitative RT-PCR primer sequences

Supplementary Figure S1-S4

Full Western blot images belonging to Figure 2

Full Western blot images belonging to Figure 5

Supplementary source data related to Figures: See Mendeley Data, http://dx.doi.org/10.17632/s7j3gkd58p.1

Supplementary methods

Medulloblastoma samples, peptide phosphorylation arrays and gene expression profiling

Tumor tissue was obtained from n=50 untreated primary medulloblastoma specimens at diagnosis from three Dutch university medical centers (University Medical Center Groningen, Radboud University Medical Center Nijmegen, and VU University Medical Center Amsterdam) by surgical resection, as originally described in [1]. In short, surgically resected tissue specimens were snap frozen in liquid nitrogen and stored at -80°C. Patient information on gender, age at diagnosis, and subtype can be found in [1]. Local ethics committee approval was granted for use of the patient material.

Primary medulloblastoma samples were analyzed for phosphoprotein-signaling previously as described in the (supplemental) materials and methods section of [1]. In short, Serine/Threonine peptide phosphorylation profiles were determined from n=50 primary medulloblastoma samples using the commercially available PamChip serine/threonine (STK) kinase microarray system (PamGene). Median signal intensities over the triplicates were normalized using the VSN method.

Medulloblastoma gene expression profiles were determined using the Illumina Human HT-12 expression beadchip system (Illumina, San Diego, USA) previously, as described in [1]. Data were quantile normalized, followed by supervised hierarchical clustering analysis using a variance filter of 0.25 and p-value <0.005. Pathway enrichment analysis was performed by uploading lists of up- or downregulated genes of pCREB Ser$^{133/lo}$, *CREBBP*^{lo} and *EP300*^{lo} medulloblastoma samples to the String database for protein-protein interaction networks, and analyzed for gene ontology of biological processes.

Quantitative RT-PCR

mRNA from P7 CGNP cultures was isolated using the RNeasy Mini Kit (Qiagen) according to the manufacturer's instructions. 500 ng RNA was used for reverse transcription with random hexamer primers and the RevertAid kit (Invitrogen). Quantitative RT-PCR was performed on a LightCycler 480 II Real-time system (Roche) using Universal SYBRGreen Supermix (BioRad). Relative gene expression was calculated using the $2^{-\Delta\Delta Ct}$ method. Expression levels are normalized to housekeeping gene Gapdh. Primers are summarized in Supplementary Table S3.

Supplementary Table S2: Short hairpin RNA sequences

shCREB1.1	5'-ATACAGCTGGCTAACAATGGTA-3'
shCREB1.2	5'-AGAGAGAGGTCCGTCTAATGAA-3'
shCREBBP.1	5'-TCGCCACGTCCCTTAGTAACCA-3'
shCREBBP.2	5'-GCCGTTTACCATGAGATCCTTA-3'
shEP300	5'-TACTCAGCCGGAGGATATTTCA-3'

Supplementary Table S3: Quantitative RT-PCR primer sequences

mDcx-F	5'-ACTGTGCTGGCGTTGAAGG-3'
mDcx-R	5'-CGGGTCCCACATTGCTTAGG-3'
mGrin1-F	5'-CCGTGAACGTGTGGAGGAA-3'
mGrin1-R	5'-CTGCTCTACCACTCTTTCTATC-3'
mGrin2a-F	5'-GGGTACATCTTTGCCACCAC-3'
mGrin2a-R	5'-CATCTCACCGTCACCAACAAAC-3'
mGrin2b-F	5'-GCCATGAACGAGACTGACCC-3'
mGrin2b-R	5'-GCTTCCTGGTCCGTGTCATC-3'
mGapdh-F	5'-AGGGCTCATGACCACAGTC-3'
mGapdh-R	5'-GATGCAGGGATGATGTTCTG-3'

Supplementary Figure S1. pCREB Ser^{133} peptide phosphorylation and *CREB1* mRNA levels in a cohort of primary medulloblastoma samples.

(a) Dot plot showing VSN normalized peptide phosphorylation intensities of pCREB Ser^{133} across medulloblastoma subgroups ($n_{SHH} = 13$, $n_{Group3} = 16$, $n_{Group4} = 19$, Tukey *post-hoc* test of one-way ANOVA). Error bars indicate mean ± 95%CI. *p<0.05, **p<0.01, **** p<0.0001. **(b)** Dot plot showing quantile normalized mRNA expression levels of *CREB1* across medulloblastoma subgroups ($n_{SHH} = 13$, $n_{Group3} = 16$, $n_{Group4} = 20$, Tukey *post-hoc* test of one-way ANOVA). Error bars indicate mean ± 95%CI. *p<0.05, **** p<0.0001.

a

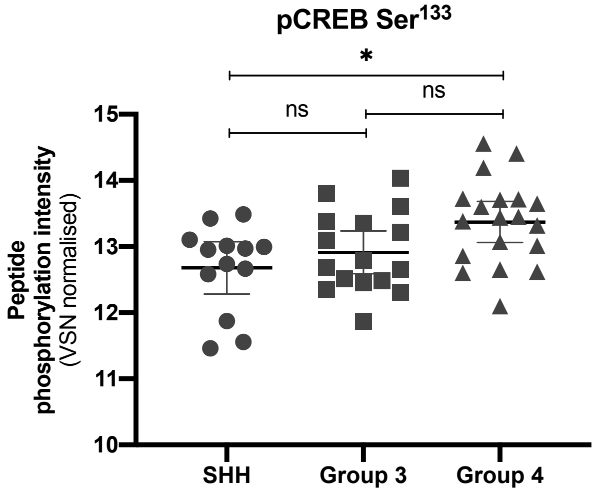

b

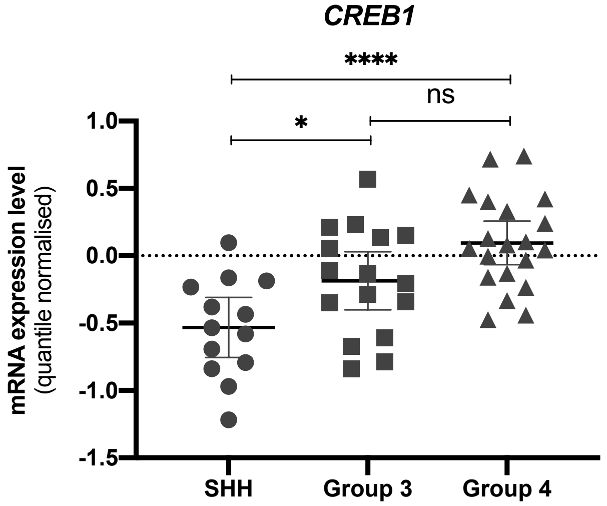

Supplementary Figure S2. *CREBBP* and *EP300* mRNA levels in primary medulloblastoma samples.

(a,b) Dot plots showing quantile normalized mRNA expression levels of (**a**) *CREBBP* and (**b**) *EP300* in primary medulloblastoma (n_{SHH} = 13, n_{Group3} = 16, n_{Group4} = 20, Tukey *post-hoc* test of one-way ANOVA). Error bars indicate mean ± 95% CI.

a

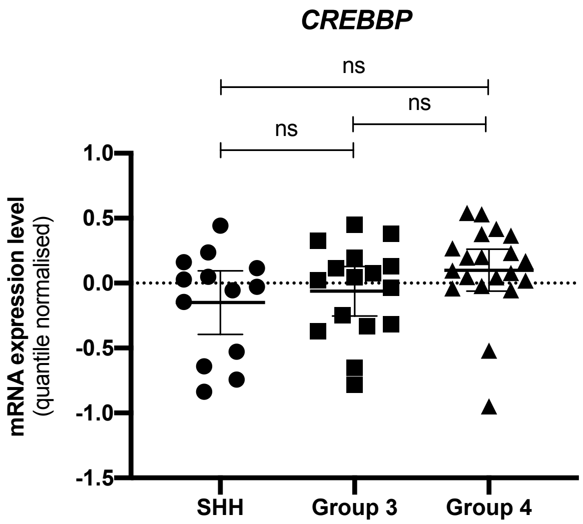

b

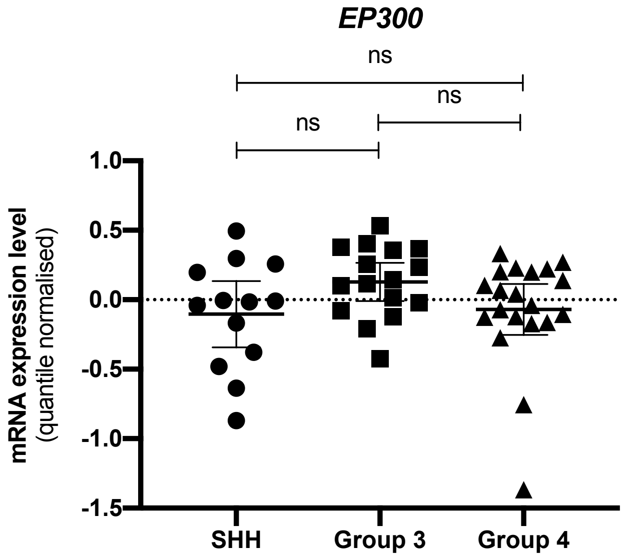

Supplementary Figure S3. mRNA expression levels of differentiation genes in primary medulloblastoma samples.

Dot plots showing quantile normalized mRNA expression levels of (**a**) *ZIC2*, (**b**) *GRIN2A*, and (**c**) *GRIN2C* in primary medulloblastoma (n_{SHH} = 13, n_{Group3} = 16, n_{Group4} = 20, Tukey *post-hoc* test of one-way ANOVA). Color codes: blue=pCREB Ser$^{133\ lo}$/*CREBBP*^{lo}/*EP300*^{lo}; red=pCREB Ser$^{133\ hi}$/*CREBBP*^{hi}/*EP300*^{hi}; black=intermediate. Error bars indicate mean ± 95% CI. *p<0.05, **p<0.01, **** p<0.0001

a

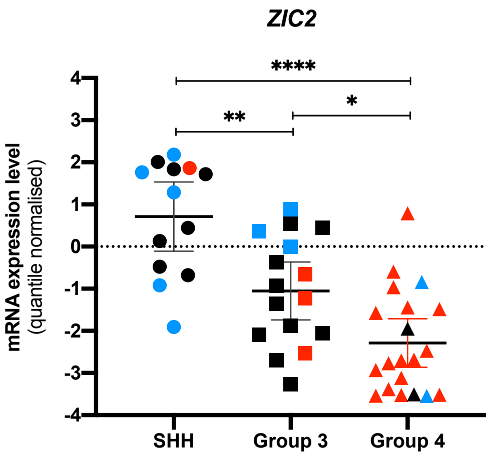

b

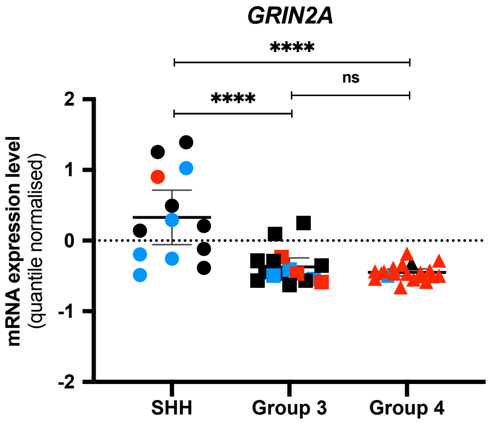

c

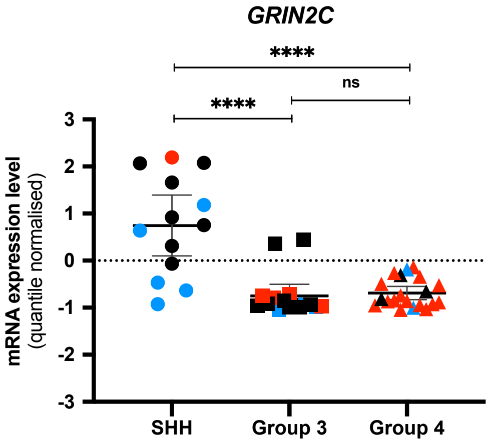

Supplementary Figure S4. Quantification of (phospho-)CREB and (phosphor-)ERK protein expression after Forskolin, Bmp6, or Bmp12 treatment in serum starved RPE-1 cells.

(a) Bar plots showing protein expression levels of (**a**) phospho-CREB and total CREB in serum starved RPE-1 following treatment with FSK (5 µM), Bmp6 (250 ng/mL), Bmp12 (250 ng/mL), or control in the presence or absence of PKA pathway inhibitor H89 (20 µM) (n=4, unpaired t-test); **(b,c)** Bar plots showing protein expression levels of **(b)** phospho-CREB and total CREB and **(c)** phospho-ERK and total ERK in serum starved RPE-1 following treatment with FSK (5 µM), Bmp6 (250 ng/mL), Bmp12 (250 ng/mL), or control in the presence or absence of MEK/ERK pathway using MEK inhibitor, PD98059 (25 µM) (n=3, unpaired t-test). Results represent mean ± SEM. *p<0.05, **p<0.01, ***p<0.001.

a

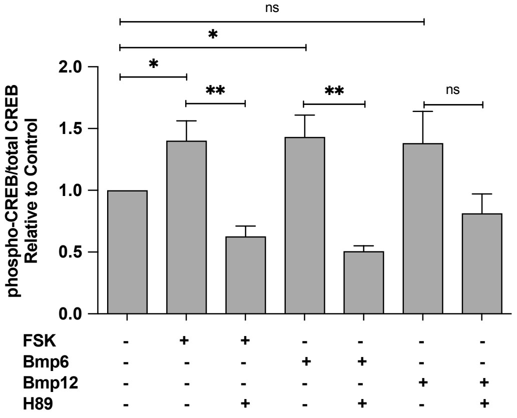

b

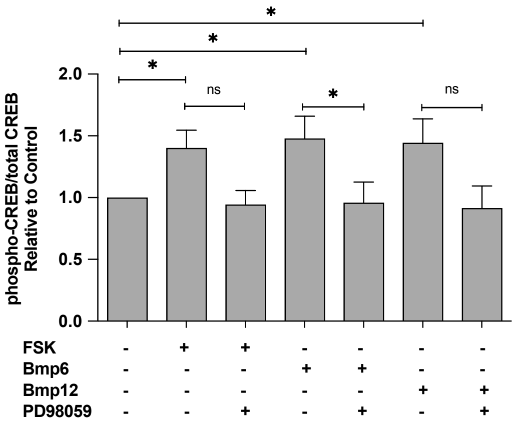

c

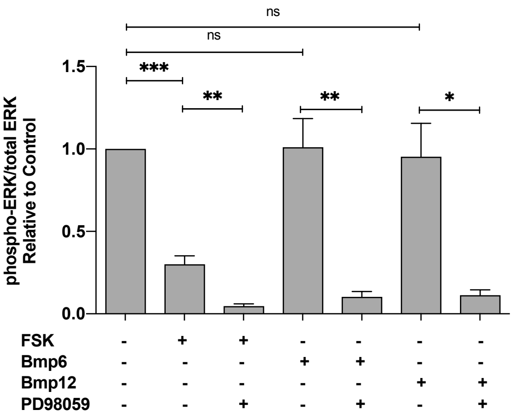

Full Western blot images belonging to Figure 2

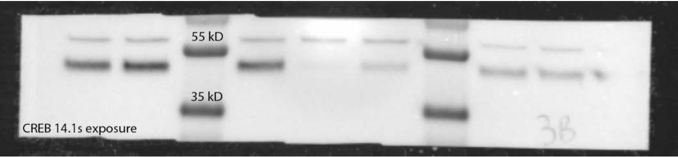
55 kD
35 kD
CREB 14.1s exposure
3B

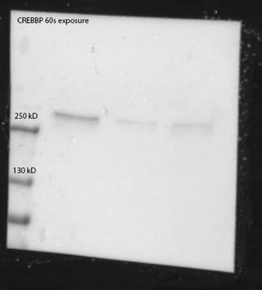
CREBBP 60s exposure
250 kD
130 kD

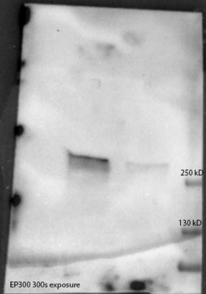
250 kD
130 kD
EP300 300s exposure

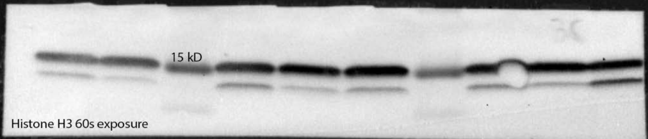
15 kD
Histone H3 60s exposure

Full Western blot images belonging to Figure 5

Fig5A.1-2 CREB 4.1sec exposure

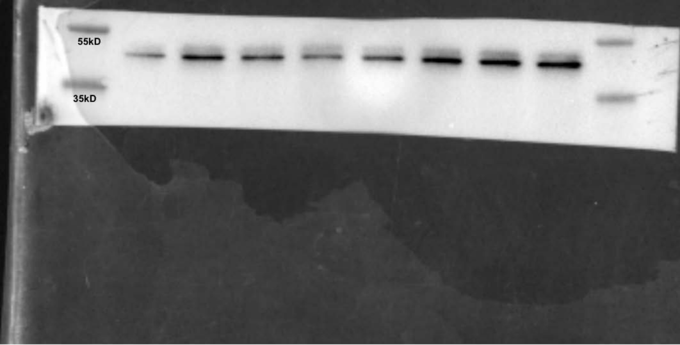

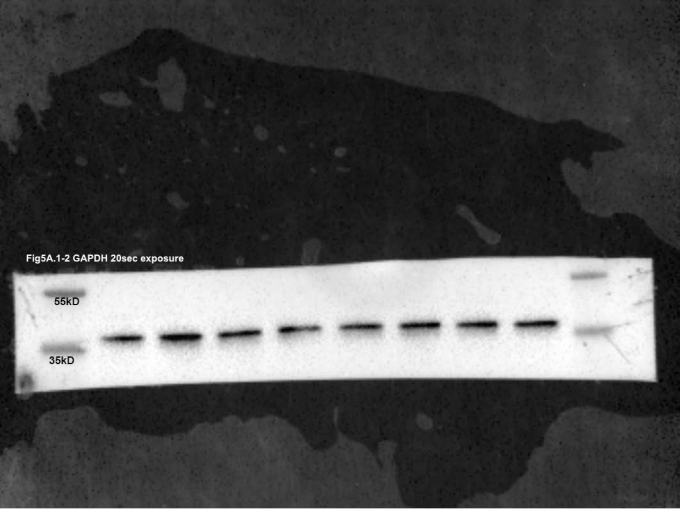
Fig5A.1-2 GAPDH 20sec exposure
55kD
35kD

Fig5A.1-2 pCREB 10sec exposure

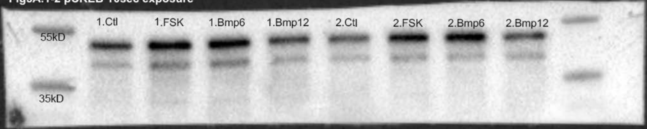

Fig5A.3 CREB 3sec exposure

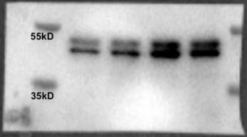

Fig5A.3 GAPDH 0.75sec exposure

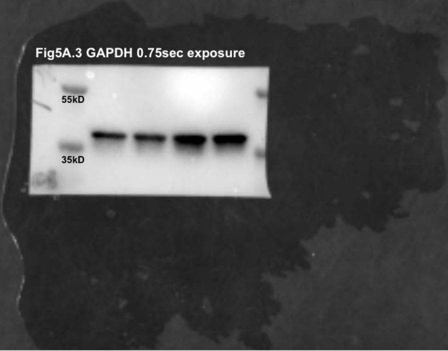

Fig5A.3 pCREB 1.5sec exposure

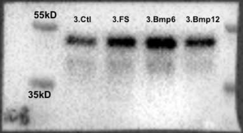

Fig5B.1 CREB 1.5sec exposure

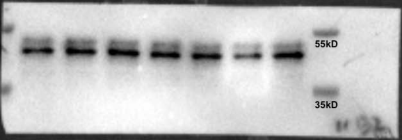

Fig5B.1 GAPDH 0.5sec exposure

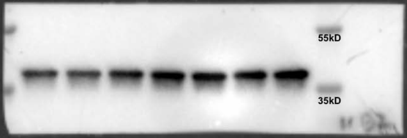

Fig5B.1 pCREB 11sec exposure

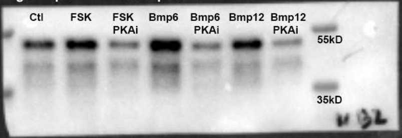

Fig5B.2 CREB 10sec exposure

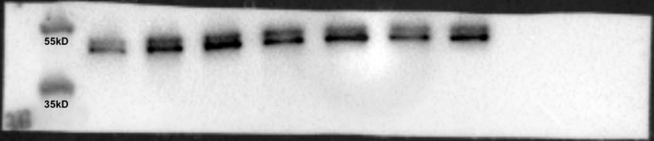

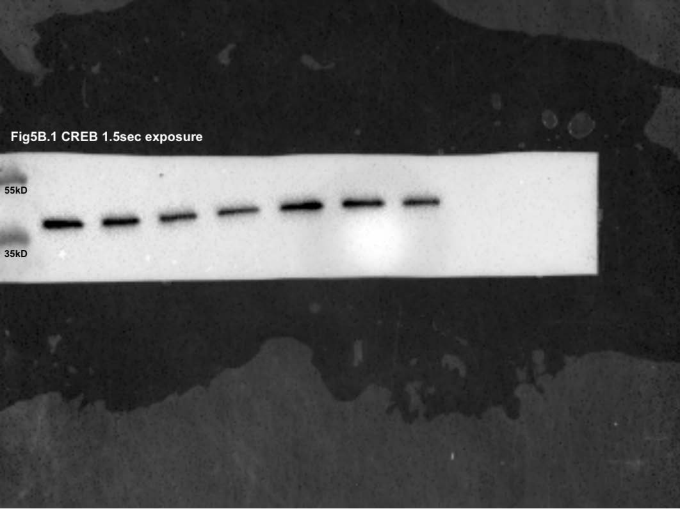
Fig5B.1 CREB 1.5sec exposure
55kD
35kD

Fig5B. p-CREB 10s exposure

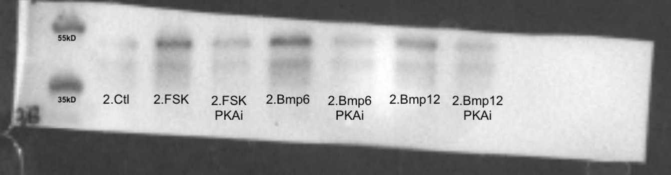

Fig5B.1 CREB 1.5sec exposure Fig5B.3 CREB 8sec exposure

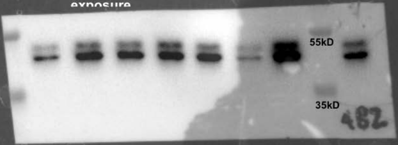

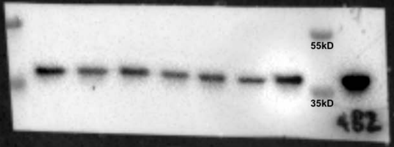

Fig5B.3 GAPDH 20sec exposure

Fig5B.3 pCREB 10sec exposure

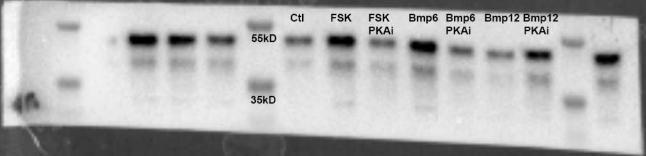

Fig5C.1A CREB 3sec exposure

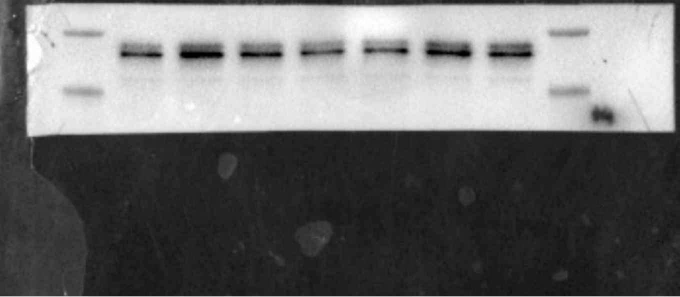

Fig5C.1A GAPDH 7sec exposure

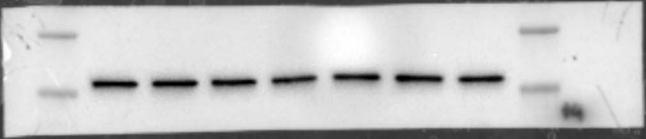

Fig5C.1A pCREB 25sec exposure

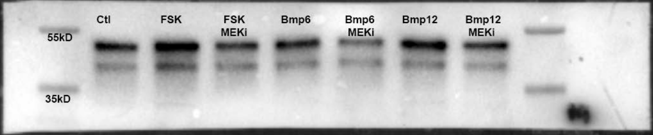

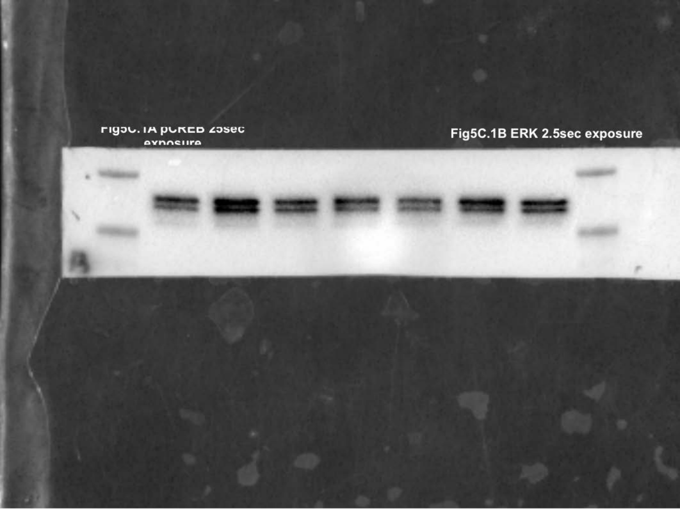
Fig5C.1A pCREB 25sec exposure
Fig5C.1B ERK 2.5sec exposure

Fig5C.1B GAPDH 4sec exposure

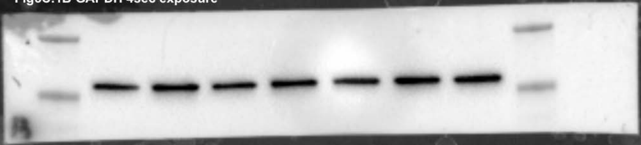

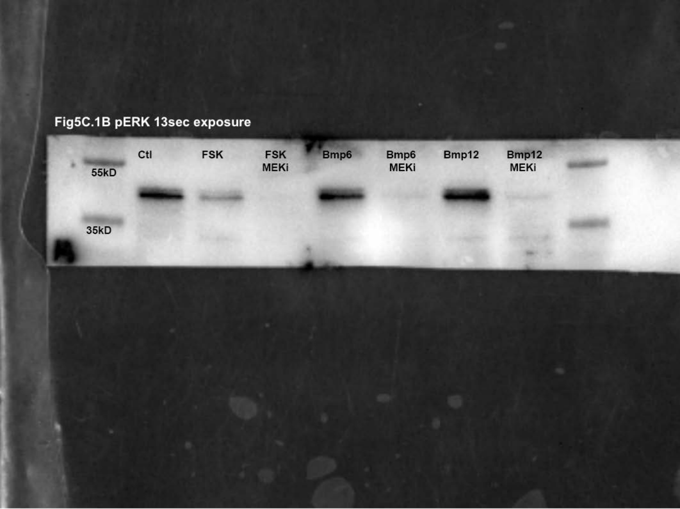
Fig5C.1B pERK 13sec exposure
55kD
35kD
Ctl
FSK
FSK MEKi
Bmp6
Bmp6 MEKi
Bmp12
Bmp12 MEKi

Fig5C.2A CREB 2.5sec exposure

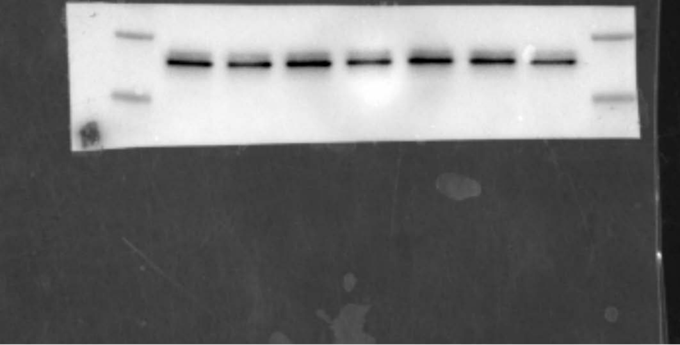

Fig5C.2A GAPDH 0.75sec exposure

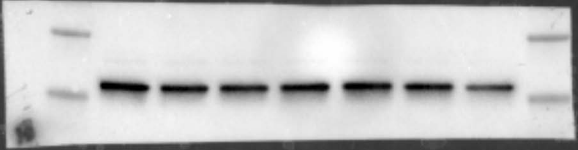

Fig5C.2A pCREB 8sec exposure

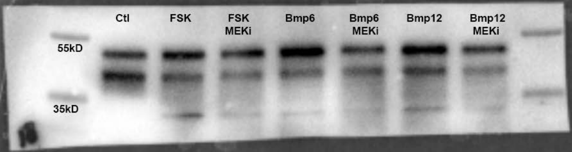

Fig5C.2B ERK 7sec exposure

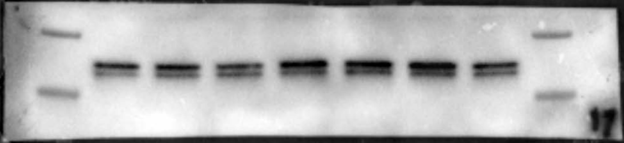

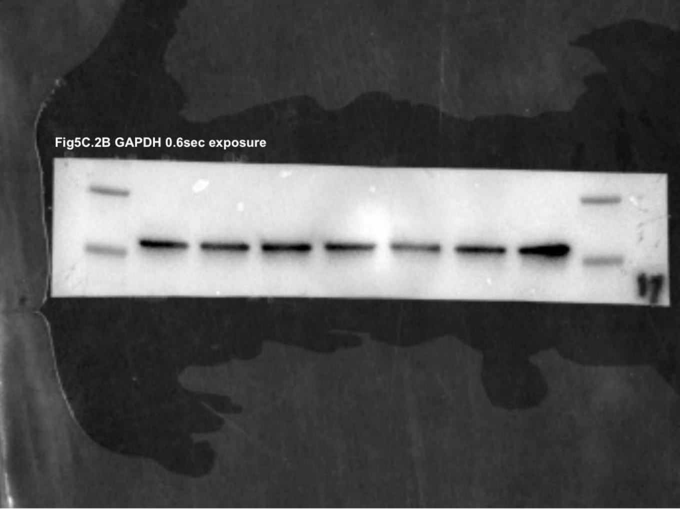

Fig5C.2B GAPDH 0.6sec exposure

Fig5C.2B pERK 4sec exposure

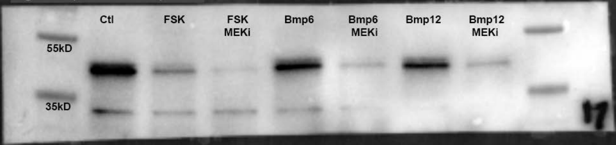

Fig5C.3A CREB 15sec exposure

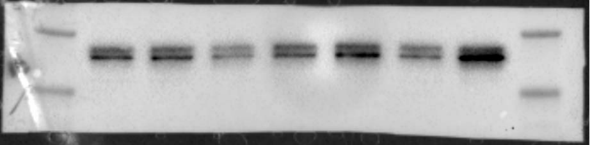

Fig5C.3A GAPDH 19sec exposure

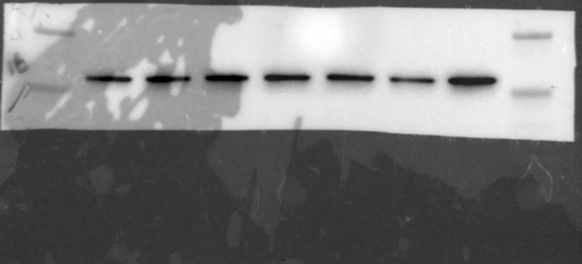

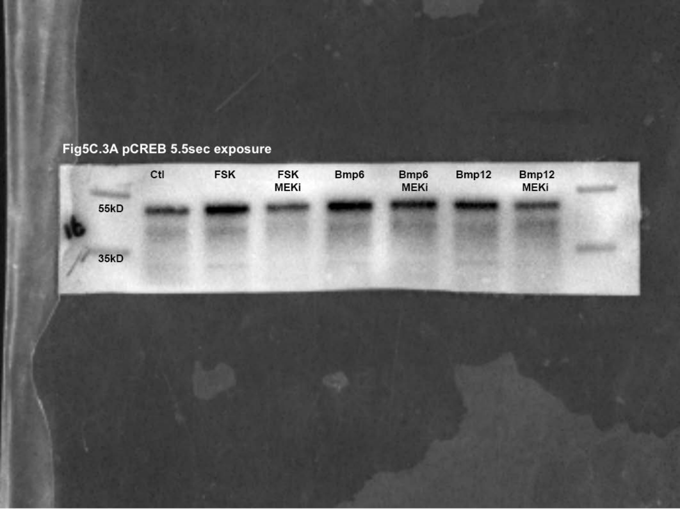
Fig5C.3A pCREB 5.5sec exposure
Ctl
FSK
FSK MEKi
Bmp6
Bmp6 MEKi
Bmp12
Bmp12 MEKi
55kD
35kD

Fig5C.3B ERK 36sec exposure

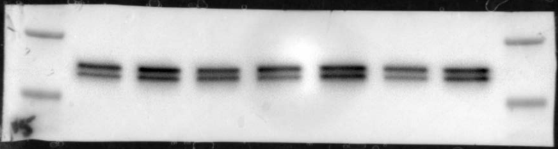

Fig5C.3B GAPDH 19sec exposure

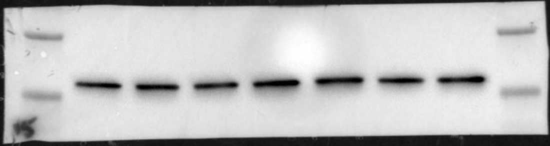

Fig5C.3B pERK 4.5sec exposure

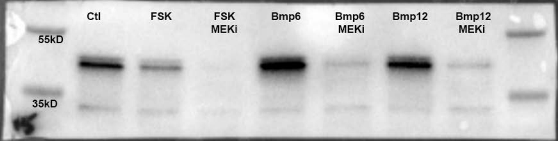

Fig5E. UW426 CREB 3sec exposure

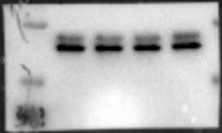

Fig5C.1A pCREB 25sec exposure

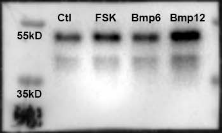

References

1. Zomerman WW, Plasschaert SLA, Conroy S, et al (2018) Identification of Two Protein-Signaling States Delineating Transcriptionally Heterogeneous Human Medulloblastoma. Cell Rep. https://doi.org/10.1016/j.celrep.2018.02.089
